# A Role for Parasites in Stabilising the Fig-Pollinator Mutualism

**DOI:** 10.1371/journal.pbio.0060059

**Published:** 2008-03-11

**Authors:** Derek W Dunn, Simon T Segar, Jo Ridley, Ruth Chan, Ross H Crozier, Douglas W Yu, James M Cook

**Affiliations:** 1 Division of Biology, Imperial College London, Ascot, United Kingdom; 2 School of Biological Sciences, University of Reading, Reading, United Kingdom; 3 School of Biological Sciences, University of East Anglia, Norwich, United Kingdom; 4 School of Marine and Tropical Biology, James Cook University, Townsville, Queensland, Australia; 5 Natural Environment Research Council (NERC) Centre for Population Biology, Imperial College London, Ascot, United Kingdom; Cornell University, United States of America

## Abstract

Mutualisms are interspecific interactions in which both players benefit. Explaining their maintenance is problematic, because cheaters should outcompete cooperative conspecifics, leading to mutualism instability. Monoecious figs (*Ficus*) are pollinated by host-specific wasps (Agaonidae), whose larvae gall ovules in their “fruits” (syconia). Female pollinating wasps oviposit directly into *Ficus* ovules from inside the receptive syconium. Across *Ficus* species, there is a widely documented segregation of pollinator galls in inner ovules and seeds in outer ovules. This pattern suggests that wasps avoid, or are prevented from ovipositing into, outer ovules, and this results in mutualism stability. However, the mechanisms preventing wasps from exploiting outer ovules remain unknown. We report that in Ficus rubiginosa, offspring in outer ovules are vulnerable to attack by parasitic wasps that oviposit from outside the syconium. Parasitism risk decreases towards the centre of the syconium, where inner ovules provide enemy-free space for pollinator offspring. We suggest that the resulting gradient in offspring viability is likely to contribute to selection on pollinators to avoid outer ovules, and by forcing wasps to focus on a subset of ovules, reduces their galling rates**.** This previously unidentified mechanism may therefore contribute to mutualism persistence independent of additional factors that invoke plant defences against pollinator oviposition, or physiological constraints on pollinators that prevent oviposition in all available ovules.

## Author Summary

Much biodiversity ultimately relies on cooperation between different species, interactions called mutualisms. Benefits to one partner are gained by obtaining resources from the other, presenting a problem: what prevents one partner from exploiting the other at an unsustainable level? Fig trees are pollinated by tiny wasps that only develop successfully themselves by each destroying a single female fig flower that would otherwise become a seed. Wasps tend to occur in long flowers near the fruit's centre, with seeds developing near the outer wall. Female wasps therefore favour long flowers for their offspring, and leave short flowers to develop into seeds. To understand why wasps exploit fig trees sustainably, we need to explain why this preference has evolved. The fig-pollinator mutualism is exploited by small parasitic wasps that attack pollinators from outside the fruit. In three Australasian fig species, we found that pollinator offspring in the outer layer of flowers were more likely to be parasitized than those in the inner layer. Our data thus indicate that long flowers provide enemy-free space for pollinator offspring at the fruit's centre. We suggest that the provision of variable length flowers by fig trees may contribute to mutualism stability by indirectly involving a third party: parasitic wasps, previously regarded as detrimental to both mutualists.

## Abstract 3

The larvae of pollinating wasps in the inner flowers of figs are safe from parasitic wasps. Parasites may contribute to stability in the fig-pollinator mutualism because outer flowers avoided by pollinators tend to develop into seeds.

## Introduction

In a biosphere driven by selection at the level of the individual gene [1], explaining the existence of cooperation, such as mutualism, is a major scientific challenge. Mutualisms are interspecific ecological interactions characterised by reciprocal benefits to both partners [2] that usually involve costly investments by each. What factors thus prevent one partner from imposing unsustainable costs onto the other to enable mutualism stability [3–7]? In some mutualisms, the larger, more sessile partner, manipulates the other by directing benefits to cooperative individuals and costs to cheaters [4–7]. However, a general consensus on mutualism persistence has only recently been formulated, and this clearly shows that a high benefit-to-cost ratio of cooperating is one important factor [8,9].

Fig trees (*Ficus*) and their host-specific agaonid pollinator wasps are a classic example of an obligate mutualism [10,11]. The wasps pollinate the trees, and the trees provide resources for wasp offspring. In monoecious *Ficus*, female wasps push their way through a specialised entrance into receptive syconia (colloquially, “figs”), which are enclosed inflorescences. The wasps then pollinate the tree while depositing their eggs individually into ovules. Thus, each egg laid costs the tree one seed, but upon emergence, the female wasp offspring disperse that tree's pollen. Trees need to produce both wasps and seeds for the mutualism to persist, but natural selection should favour wasps that exploit the maximum number of fig ovules in the short term, resulting in a conflict of interest between wasp and tree. However, the mutualism has persisted for at least 60 million years and has radiated into more than 750 species pairs [12]. The mechanisms preventing wasps from overexploiting figs remain unknown, despite intensive study over four decades.

**Figure 1:**
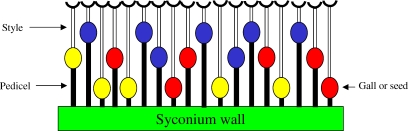
The Spatial Stratification of Pollinators, Parasites, and Seeds in the F. rubiginosa Syconium Blue galls contain pollinators, red galls contain parasites, and yellow ovules contain seeds. “Ovule length” is used to estimate the distance of the seed or gall from the syconium wall and is measured as the point the pedicel joins the fig wall to the top of the seed or gall, excluding what remained of the style.

Within receptive syconia, the lengths of floral styles are highly variable [13,14], and ovipositing pollinators (foundresses) favour flowers with shorter styles for their offspring [15–18]. Style and pedicel lengths of flowers are negatively correlated. Short-styled ovules develop into seeds or galls (when a wasp is present) near the syconium inner cavity, while most long-styled ovules develop into seeds near the outer wall [19,20] (Figure 1). These patterns have been shown to reflect the oviposition preferences of foundresses, and are unlikely to be the result of greater elongation of pedicels containing eggs during syconial maturation, because in receptive syconia, pollinators' eggs are mainly present in short-styled inner ovules [16]. These widespread observations have been tied to four, not necessarily mutually exclusive, mechanisms that have been proposed to stabilise the fig-pollinator mutualism: (1) Unbeatable seeds—outer ovules may be defended biochemically or physically against oviposition or larval development [21]. However, no mechanism has yet been identified. (2) Short ovipositors—pollinators' ovipositors may be too short to fully penetrate the long styles of outer ovules [14,19]. However, many pollinator wasp species have ovipositors that are long enough to reach most or all ovules [18,19,22]. (3) Insufficient eggs—because pollinators disperse passively over long distances, too few foundresses may arrive to fill all ovules, and they fill inner ovules first because these are likely to be easier for oviposition [19]. Alternatively, the tree may limit the number of foundresses that enter its syconia [20]. However, in the majority of *Ficus* species, syconia receive enough foundresses to exploit more ovules than in fact produce wasps, leaving a large proportion of seed production unexplained [23,24]. Consequently, these three hypotheses have failed to explain mutualism stability in monoecious *Ficus* species, but all suggest that the key to the puzzle lies in explaining why pollinating wasps favour inner ovules for oviposition.

Recently, a fourth hypothesis, based on “optimal foraging” by ovipositing foundresses, has been proposed. Simulations have shown that the fig-pollinator mutualism can be stabilised if ovule profitability is correlated with flower style length, and if some foundresses die before laying all of their eggs [24]. The profitability of an ovule to a foundress depends on the expected offspring fitness divided by the handling time needed to lay an egg. Foundresses are thus expected to prefer short-styled flowers (inner ovules) if handling times to enable successful oviposition are lower and/or inner ovules yield higher offspring fitness than outer ovules. Therefore, the greater the relative profitability of inner ovules, the more that foundresses are likely to be selected to spend their short lives searching for them, even as inner ovules become rare. Indeed, when foundress numbers are experimentally controlled, an increased number of foundresses has been shown to result in a higher proportion of exploited inner ovules within a syconium, rather than in the total number of ovules per se [16]. Thus, the predicted consequence to a foundress in a syconium already full of exploited inner ovules is reduced fitness, with seed production in outer ovules being protected because some foundresses are likely to die before exploiting all nonpreferred outer ovules [24].

Although crucial in determining foundress behaviour, the fitness differential for wasp larvae developing in inner versus outer ovules is largely unknown. However, there is evidence that inner ovules develop into larger galls due to increased space near the syconium cavity (the lumen), resulting possibly in larger, more fecund female offspring [15]. Additionally, the fig-pollinator mutualism is ubiquitously exploited by a suite of nonpollinating wasps [10,11,25], which could also alter the relative values of ovules to foundresses. This is because many nonpollinating fig wasps are parasites (parasitoids or inquilines) that insert their ovipositors into the syconium from the outside and deposit parasite offspring that kill pollinator larvae already present in galls [10,11,25,26]. It is widely perceived that nonpollinating fig wasps compete with pollinators for inner ovules [22,27] and have negative effects on the fig by reducing the production of pollinator offspring [3,11,12,25]. However, the relative positions of parasitized and unparasitized pollinators within the same syconium have never been directly and precisely measured. If parasites are more likely to parasitize pollinator offspring in the outer layers of ovules, this will increase the fitness value of inner ovules to foundress pollinators because their offspring will have increased survivorship in the “enemy-free space” at the centre of a syconium. Thus, parasitic fig wasps could make a contribution to the maintenance of the fig-pollinator mutualism, by being one of the selection pressures that have resulted in foundresses favouring inner ovules.

## Results/Discussion

**Figure 2:**
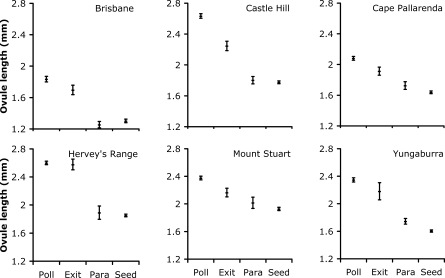
The Mean Lengths (±s.e.) of Ovules Containing Seeds, Parasites, Pollinators, or Those That Had Been Exited by Males, within F. rubiginosa Syconia from Six Queensland Sites Controlling for variance in ovule length that can be attributed to the difference between sites (*F*_5,5856_ = 110.96, *p* < 0.001) and syconium size (*F*_1,5856_= 34.64, *p* < 0.001), the differences in means between the four ovule categories is highly significant (*F*_3,5856_ = 622.00, *p* < 0.001). Because we had a priori expectations prior to data collection, we used contrast analysis instead of post hoc tests to measure differences in means between categories. Ovules containing pollinators (Poll) were significantly longer than the other three categories (*p* < 0.001 for all three tests). Exit, exited; Para, parasites.

**Figure 3:**
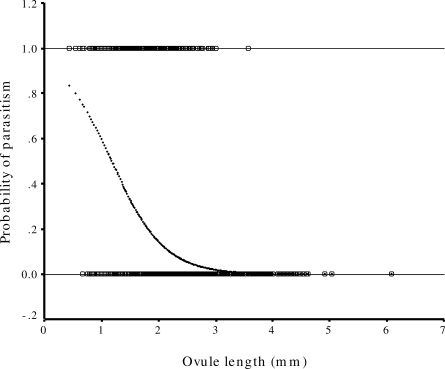
The Probability of Parasitism as a Function of Ovule Length Small, filled symbols represent the fitted binary logistic regression model, which used parasite or pollinator presence as the dependent variable (large, open symbols).

If inner ovules represent enemy-free space for pollinator larvae, we would predict that externally ovipositing parasitic wasps are more likely to kill pollinator larvae in outer ovules. In the syconia of F. rubiginosa, collected from six sites in Queensland, Australia, seeds, parasites, and pollinators were spatially stratified in the same order. Inner ovules were significantly more likely to contain pollinators, and outer ovules, seeds or parasites. The ovules already exited by male wasps (a combination of pollinators and parasites) were intermediate in length between those still containing pollinators and those still containing parasites (Figure 2). While controlling for variation in parasitism rates between sites (Wald = 112.05, *p* < 0.001), we found that the risk of parasitism to a pollinator offspring decreased significantly, from 80% nearest the fig wall to 0% toward the centre of the syconium (β ± standard error [s.e.] = −2.21 ± 0.14, Wald = 240.16, *p* < 0.001; Figure 3). Ovule profitability to foundresses, measured as offspring survivorship, therefore shows a strong increase from the wall to the central cavity of a syconium, even before counting any reduced time required to oviposit in inner ovules. In addition, the overall level of parasitism decreases as syconia get larger (β ± s.e. = −0.002 ± 0.00, Wald = 33.41, *p* < 0.001), supporting our hypothesis that parasites are limited in their ability to reach ovules farthest from the syconium wall. Moreover, across syconia, the mean length of parasite-occupied ovules positively correlates with the mean length of those occupied by pollinators (*F*_1,45_ = 4.85, *p* = 0.033), independent of the effects of site (*F*_5,45_ = 17.22, *p* < 0.001) and syconium size (*F*_1,45_ = 6.66, *p* = 0.013) (longer ovules are further from the syconium wall). This variation probably reflects variation in gall and pedicel elongation during maturation and further shows that parasites consistently fail to attack inner ovules.

**Figure 4:**
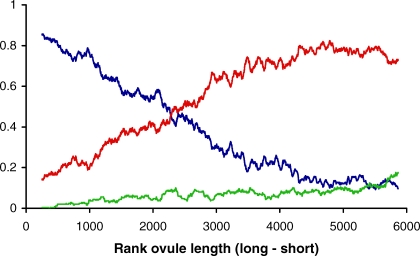
The Frequency of Occurrence of Pollinators, Parasites and Seeds, in Galls Ranked for Length across Our Complete Dataset Long galls (inner ovules) are on the left, and short galls (outer ovules) are on the right of the *x*-axis. The red line indicates seeds, blue indicates pollinators, and green indicates parasites. Frequencies are moving averages over intervals of 250 ovules (i.e., frequencies for ovules ranked 1–249, 2–250, 3–251…).

Over 90% of the nonpollinating wasps we found belonged to two genera, *Philotrypesis* and *Sycoscapter* [28]. Individuals of these genera develop at the expense of a pollinator offspring, either by consuming the pollinator larva directly (parasitoid) or by killing the larva and consuming the developing seed tissue (inquiline). The occurrence of parasites in the outermost ovules demonstrates the prior presence of pollinators (Figure 3), and thus, the spatial stratification of seeds and pollinators in our dataset cannot be explained by all outer ovules being “unbeatable” for pollinators [21] or by the ovipositors of pollinators being too short to reach outer ovules [14,19]. It should be noted, however, that the spatial patterns of pollinators, parasites, and seeds in our data do not eliminate the possibility that a subset of outer ovules might still be unavailable to foundresses for unknown reasons. Additionally, we ranked all ovules across our dataset for length, and then plotted the frequency of occurrence for three categories: seeds, pollinators, and parasites. Although the frequency of parasitism varies considerably in our dataset (Table 1), there is a clear negative relationship between parasite and pollinator presence, and an increase in the likelihood of seed development, as galls get shorter (Figure 4), suggesting that parasite presence contributes to the overall factors that prevent pollinators exploiting outer ovules to enable the trees to produce seeds.

**Table 1:**
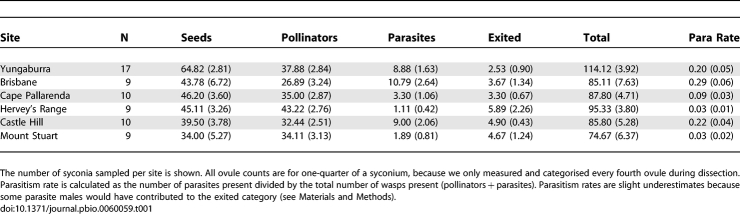
Mean Frequencies (±s.e.) of the Four Categories of Ovules Present in F. rubiginosa Syconia from Six Queensland Sites (See Materials and Methods for Ovule Categorisation Criteria), the Mean Total Number of Ovules, and Mean Parasitism Rate per Syconium

Egg limitation in the pollinator, Pleistodontes imperialis, is unlikely to contribute to the stability of its mutualism with F. rubiginosa. Two foundresses carry enough eggs (mean eggs per foundress ± s.e. = 231.58 ± 12.53, *N* = 36) to exploit all ovules in a syconium (mean ovules per syconium ± s.e. = 373.25 ± 86.43, *N* = 64). The mean number of foundress bodies (mean ± s.e. = 2.58 ± 0.12, *N* = 203) clearly shows that the amount of wasp eggs that enter an F. rubiginosa syconium exceeds the number of ovules (D. W. Dunn, S. Al-beidh, C. Reuter, S. T. Segar, D. W. Yu, J. Ridley, and J. M. Cook, unpublished data).

**Figure 5:**
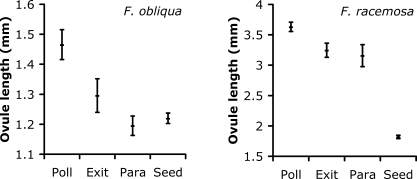
The Mean Lengths (±s.e.) of Ovules Containing Seeds, Parasites, Pollinators, or Those That Had Been Exited by Males in F. obliqua and F. racemosa Data from three sites per species have been pooled for ease of comparison. Exit, exited; Para, parasites; Poll, pollinators.

The contribution parasitic wasps may make to the overall mechanisms that lead to mutualism stability across *Ficus* is clearly likely to vary across such a speciose and variable genus [12,22]. A comprehensive taxonomic investigation is clearly beyond the scope of this study. However, within their natural geographic ranges, the larvae of pollinators across monoecious *Ficus* are likely to be subject to attack by externally ovipositing parasitic wasps (e.g., [11,25]). Moreover, the syconia across *Ficus* species are highly variable in size. In smaller species, there may thus be physical constraints on the spatial segregation of pollinators and parasites. To test whether our parasite pressure hypothesis is likely to be restricted to the Malvanthera section within *Ficus*, or if syconium size is likely to constrain spatial segregation of pollinators and parasites, we studied two additional monoecious *Ficus* species. Ficus obliqua is a close relative of F. rubiginosa [29] but has small syconia (mean diameter at wasp emergence ± s.e. = 6.57 ± 0.34 mm, *N* = 16). In contrast, F. racemosa has large syconia (mean diameter at wasp emergence ± s.e. = 26.17 ± 0.75 mm, *N* = 14) and also belongs to a distantly related clade of figs, the vast majority of which are dioecious [30]. It therefore represents a different origin of monoecy to Malvantheran *Ficus*. In both of these additional species, we found similar spatial stratification of pollinators and parasites as in F. rubiginosa (Figure 5; Text S1), suggesting that a potential contribution of parasitic fig wasps to the overall factors that enable stability in the fig-pollinator mutualism is neither lineage specific, nor limited by small syconium size.

Finally, we found that pollinator body size did not correlate with ovule length (*F*_1,55_ = 0.44, *p* = 0.51), although there was considerable between-site variance (*F*_5,55_ = 13.19, *p* < 0.001). Larger syconia tended to contain larger female pollinators (*F*_1,55_ = 6.75, *p* = 0.012), but the relationship was curvilinear, such that the largest syconia contained smaller pollinators (second-order term: *F*_1,55_ = 6.69, *p* = 0.012). Thus, we found no evidence that foundresses may select inner ovules for benefits associated with producing large offspring, although there may be benefits in entering a syconium of intermediate size.

Our study is the first to show that pollinating fig wasps may gain a fitness benefit by selecting inner ovules for oviposition, because these ovules have reduced vulnerability to parasitism. The provision of ovules with high variance in profitability to foundresses clearly demonstrates that the larger, more sessile partner in the symbiosis [7], the fig tree, controls the resources available to the smaller, more mobile partner. Selection could benefit those trees producing syconia that are partially vulnerable to parasitism, via selection on the toughness and thickness of syconial walls and/or variation of floral style, and hence pedicel, lengths (Figure 1). This variance in floral morphology, and the strong likelihood of the occurrence of externally ovipositing parasitic fig wasps across monoecious *Ficus*, indicates a wide-ranging potential for parasitic wasps to contribute to stability in the fig-pollinator mutualism. At the smaller scale, this variable floral environment is likely to give a fitness advantage to the first foundresses to enter a receptive syconium by “providing” an abundance of safe inner ovules in which to deposit their offspring. Later foundresses, who will carry pollen of a lower value to the tree because early foundresses will have already distributed the pollen they carried, are thus effectively “penalised” for exploiting outer ovules. Our data thus show that the benefits to foundresses exploiting outer ovules are reduced by the parasitism costs to offspring, and demonstrate how a third party may select for more beneficial behaviour in a symbiont.

The potential role played by parasitic wasps may also help to resolve the evolutionary paradox posed by fig trees having generation times several orders of magnitude longer than those of their pollinators [12,22]. Presumably, a coevolutionary arms race should be resolved in favour of the pollinator, but not if a gradient in ovule profitability is produced in part by exposure to parasitic wasps, which have similar generation times to pollinators. However, the inner ovules used favourably by pollinator wasps provide an untapped resource for parasites, and one would expect strong selection for longer ovipositors in parasites to enable the exploitation of more hosts. We suggest, however, that relatively long ovipositors will have costs to the individual parasitic wasps as well as benefits. For instance, the aerodynamic influences on flight will change with a relatively long ovipositor. Likewise, the time taken to insert the ovipositor when searching for a host is likely to increase with ovipositor length, which may lead to an increased risk of predation by ants [31]. If the costs of a long ovipositor outweigh the benefits, then net selection will not favour the evolution of very long ovipositors in all parasites.

Thus, despite the short-term costs posed by parasitic wasps to the mutualists [10,21,25], parasitic wasps may also contribute to the long-term stability of the mutualism between F. rubiginosa and its pollinator P. imperialis. Moreover, we provide evidence to suggest that parasitic fig wasps have the potential to contribute positively to the overall mechanisms that enable the fig-pollinator mutualism to remain stable in other monoecious *Ficus* species. Although the larger partner, the fig tree, clearly controls resource availability to its pollinator, our data suggest this may be realised in part by indirectly involving parasitic wasps. Our results therefore provide another example of how a third party can shift a symbiosis towards a more mutually cooperative outcome [4,32,33]. Further studies of diverse fig species should help to confirm both the generality of parasite selection pressure and test for the presence of other mechanisms [17,22] in maintaining the fig-pollinator mutualism.

## Materials and Methods

We measured both the probability of offspring mortality through parasitism, and the body sizes of female offspring, in relation to ovule position within the syconium. We used a total of 64 syconia from six populations of the Australian fig F. rubiginosa (section Malvanthera) ranging across 1,700 km of Eastern Queensland, Australia. Nine to 17 syconia were collected from a single crop from each tree. Each tree originated from a different population. Three trees (Cape Pallarenda, Castle Hill, and Mount Stuart) were from the Townsville region of northern Queensland. The other trees sampled were from Hervey's Range (50 km west of Townsville), Yungaburra (near Cairns, far north Queensland), and Brisbane (southern Queensland). All syconia were early in the male flower phase [34] with no exit holes made by male wasps. This was to ensure that female wasps had yet to emerge from their galls. Immediately after collection, all syconia were placed in 80% ethanol.

In the laboratory, each syconium was sliced into eighths lengthways. Every ovule was then systematically removed from all sections. We measured the total length of every fourth ovule (pedicel + seed or gall, excluding what remained of the style) to the nearest 0.024 mm using an eyepiece graticule attached to a binocular microscope. We did not measure the pedicel length separately for two reasons. (1) Galls or seeds at the extreme outside wall of the syconium do not have pedicels, which would result in a series of zeros in the resulting dataset and subsequent problems with data analysis. (2) In F. rubiginosa, there is no distinct landmark where the pedicel joins the gall or seed for repeatable, accurate measurements to be taken. Moreover, although galls containing wasps have been found to differ significantly in size to seeds in other species of *Ficus* [15], we are unaware of any significant size differences in galls inhabited by the parasite genera present in this study and galls inhabited by pollinators. The level of spatial stratification between parasites and pollinators is also so pronounced that for this pattern to be an artefact of size differences between parasite and pollinator galls, a large and obvious difference, such as that of galling wasps and pollinators, would have to be apparent.

After dissection, each ovule was assigned to one of four categories: (1) seed—ovules containing seeds; (2) exited—ovules with an exit hole made by a vacated male wasp; (3) parasite—in which the ovule contained a parasitic wasp, and (4) pollinator—ovules containing pollinating wasps (P. imperialis). We did not differentiate between the four “cryptic species” of P. imperialis because genetic data are required to distinguish them [35]. It is not possible to separate galls vacated by males into either pollinators or parasites. Both wasp types therefore contributed to the exited category.

The larval biology of most species of nonpollinating fig wasps has been divided into three major ecological groupings [11,22,28]: (1) large gall-making wasps and their parasites; (2) gall-makers of similar size to the pollinators; and (3) inquilines and parasitoids of similar size to the pollinators. Group 1 wasps are rare in F. rubiginosa but can alter development substantially by causing retention of unpollinated syconia. Their large galls are immediately obvious when a fig is opened, and we excluded the few such syconia found (<5%). The nonpollinating wasps found belonged overwhelmingly to group 3, and over 90% of individuals belonged to two common genera, *Sycoscapter* and *Philotrypesis.* The remainder (<10%) were split between another group 3 parasitic wasp (Watshamiella sp.) and a gall-maker from group 2 (Eukobelea sp.). Consequently, about 95% of all nonpollinating wasps in this study were identified as inquilines or parasitoids. Only 131 (2.23%) of the 5,866 ovules measured still contained a male parasite (*N* = 79) or a male pollinator (*N* = 52).

For an estimate of the body size of female P. imperialis, we measured the length and width of the head to the nearest 0.024 mm using an eyepiece graticule fitted to a binocular microscope. As a measure of syconium size, we took the mean of the width and length of each syconium (as measured to the nearest 0.05 mm with digital calipers) and used this to calculate the volume of a sphere.

### Statistical analysis.

Unless otherwise stated, we transformed all measurements to natural logarithms to normalise the error variances. We compared the mean lengths of each of the four categories of ovules, using a general linear mixed model that included ovule category and syconium volume as predictors. Site was included as a random factor.

To test our hypothesis that parasites can only gain access to pollinators in middle and outer ovule layers, we ran a general linear mixed model that used the mean length of ovules occupied by pollinators to predict the mean length of ovules occupied by parasites. Syconium volume was included as an additional covariate to control for any effects of syconium size on ovule length, and site was again included as a random factor [21].

We used a binary logistic regression to measure the relationship between ovule length and the likelihood of parasitism. Ovules containing seeds or those that had been vacated by a male wasp were excluded from the analysis. For the dependent variable, we included those ovules known to contain either a parasitic wasp (1) or a pollinator (0). Site and syconium volume were included as additional covariates to ovule length.

We estimated the head area of female pollinating wasps (length × width). To test whether pollinators were distributed nonrandomly within syconia according to their size, we used a general linear model with head area as the dependent variable, site as a random factor, and both pollinator-occupied ovule length and fig volume as covariates.

## Supporting Information

Text S1 Testing the Spatial Stratification of Pollinators and Parasites in Other *Ficus* Species (28 KB DOC)

## Abbreviation

s.e.: standard error
