# Supplementary material for: A Role for Parasites in Stabilising the Fig-Pollinator Mutualism
Source: PLoS Biol. 2008 Mar 11;6(3):e59. doi: 10.1371/journal.pbio.0060059 (PMC2265770; doi:10.1371/journal.pbio.0060059)
Supplement: Supplementary file 1 [file pbio.0060059.sd001.doc]

We tested our pollinator-parasite spatial stratification hypothesis in two additional *Ficus* species that pose separate challenges to its potential generality. We took samples from *F. obliqua* (16 syconia) and *F. racemosa* (14 syconia) at three sites for each species around the Townsville area, northern Queensland. We used the same methods as in *F. rubiginosa* to measure the relative positions of seeds, parasites and pollinators within syconia. We found similar spatial stratification of pollinator and parasitic wasps in both species (Fig. 5). After removing variance that could be attributed to unmeasured factors between sites (*F. obliqua*: *F*_2, 741_ = 10.09, *P* < 0.001; *F. racemosa: F*_2, 1758_ = 5.00, *P* = 0.007) and syconium size (*F. obliqua*: *F*_1, 741_ = 36.95, *P* < 0.001; *F. racemosa*: *F*_1, 1758_ = 5.00, *P* = 0.026), the differences in means between the four ovule categories were significant in both species (*F. obliqua*: *F*_3, 741_ = 5.40, *P* = 0.001; *F. racemosa*: *F*_3, 1758_ = 281.59, *P* < 0.001). Contrasts showed that in *F. racemosa*, ovules containing pollinator wasps were significantly longer than the other three ovule categories (*P* < 0.01 for all three tests). In *F. obliqua*, ovules containing pollinator wasps were significantly longer than ovules containing parasites (*P* = 0.028) and seeds (*P* < 0.001) but not those from which wasps had exited their gall (*P* = 0.38).
